# Incidence of Pregnancy-Associated Cancer in Two Canadian Provinces: A Population-Based Study

**DOI:** 10.3390/ijerph18063100

**Published:** 2021-03-17

**Authors:** Amy Metcalfe, Zoe F. Cairncross, Christine M. Friedenreich, Joel G. Ray, Gregg Nelson, Deshayne B. Fell, Sarka Lisonkova, Parveen Bhatti, Carly McMorris, Khokan C. Sikdar, Lorraine Shack

**Affiliations:** 1 Department of Obstetrics and Gynecology, University of Calgary, Calgary, AB T2N 1N4, Canada; zoe.cairncross@ahs.ca (Z.F.C.); gregg.nelson@ahs.ca (G.N.); 2 Department of Medicine, University of Calgary, Calgary, AB T2N 1N4, Canada; 3 Department of Community Health Sciences, University of Calgary, Calgary, AB T2N 1N4, Canada; christine.friedenreich@ahs.ca (C.M.F.); khokan.sikdar@ahs.ca (K.C.S.); lorraine.shack@ahs.ca (L.S.); 4 Department of Oncology, University of Calgary, Calgary, AB T2N 1N4, Canada; 5 Department of Cancer Epidemiology and Prevention Research, Cancer Care Alberta, Alberta Health Services, Calgary, AB T2S 3C3, Canada; 6 Department of Medicine, University of Toronto, Toronto, ON M5S 1A1, Canada; joel.ray@unityhealth.to; 7 Department of Obstetrics and Gynecology, University of Toronto, Toronto, ON M5S 1A1, Canada; 8 SickKids Research Institute, Toronto, ON M5G 0A4, Canada; 9 School of Epidemiology and Public Health, University of Ottawa, Ottawa, ON K1N 6N5, Canada; dfell@uottawa.ca; 10 Children’s Hospital of Eastern Ontario Research Institute, Ottawa, ON K1H 5B2, Canada; 11 Department of Obstetrics and Gynecology, University of British Columbia, Vancouver, BC V6T 1Z4, Canada; slisonkova@bcchr.ca; 12 Cancer Control Research, BC Cancer, Vancouver, BC V5Z 1G1, Canada; pbhatti@bccrc.ca; 13 Werkland School of Education, University of Calgary, Calgary, AB T2N 1N4, Canada; camcmorr@ucalgary.ca; 14 Alberta Health Services, Calgary, AB T5J 3E4, Canada; 15 Cancer Research and Analytics, Cancer Control Alberta, Alberta Health Services, Calgary, AB T5J 3E4, Canada

**Keywords:** cancer incidence rate, pregnancy-associated cancer, temporal trends, epidemiology, pregnancy, obstetrics, data linkage, novel use of cancer registry

## Abstract

Pregnancy-associated cancer—that is diagnosed in pregnancy or within 365 days after delivery—is increasingly common as cancer therapy evolves and survivorship increases. This study assessed the incidence and temporal trends of pregnancy-associated cancer in Alberta and Ontario—together accounting for 50% of Canada’s entire population. Linked data from the two provincial cancer registries and health administrative data were used to ascertain new diagnoses of cancer, livebirths, stillbirths and induced abortions among women aged 18–50 years, from 2003 to 2015. The annual crude incidence rate (IR) was calculated as the number of women with a pregnancy-associated cancer per 100,000 deliveries. A nonparametric test for trend assessed for any temporal trends. In Alberta, the crude IR of pregnancy-associated cancer was 156.2 per 100,000 deliveries (95% CI 145.8–166.7), and in Ontario, the IR was 149.4 per 100,000 deliveries (95% CI 143.3–155.4). While no statistically significant temporal trend in the IR of pregnancy-associated cancer was seen in Alberta, there was a rise in Ontario (*p* = 0.01). Pregnancy-associated cancer is common enough to warrant more detailed research on maternal, pregnancy and child outcomes, especially as cancer therapies continue to evolve.

## 1. Introduction

Cancer is the second leading cause of death among Canadian women aged 25–34 years, and the leading cause of death in those aged 35–44 years [1]. Young adults with cancer are recognized as having special considerations [2]; they face a long period of survivorship, at a time when they are establishing their careers and potentially considering family planning [2]. A cancer diagnosis during this period adds further complexity if it occurs proximal to pregnancy.

Pregnancy-associated cancer includes cancers diagnosed during pregnancy or within 365 days after delivery, spontaneous pregnancy loss or induced abortion [3]. The inclusion of postpartum cancer diagnoses is important, as many of the initial symptoms of cancer (e.g., fatigue, weight gain and body aches) may mimic common pregnancy symptoms, and both women and their care providers may be reluctant to pursue radiological or invasive diagnostic procedures during pregnancy [3]. The most common types of cancer diagnosed during pregnancy include breast, cervical, melanoma and lymphoma [4]. The incidence rate (IR) of pregnancy-associated cancer reported in the literature ranges from 89.6 per 100,000 pregnancies in Denmark between 1977 and 2006 [5], to 137.3 per 100,000 pregnancies in Australia between 1994 and 2007 [3]; however, these reported IRs may vary by region and era, especially as cancer therapies evolve [6]. Currently, population-based estimates of the IR of pregnancy-associated cancer in Canada are lacking. In response, this study was undertaken to estimate the IR and temporal trends of pregnancy-associated cancer in Alberta and Ontario—together comprising 50% of Canada’s entire population.

## 2. Materials and Methods

**Figure 1:**
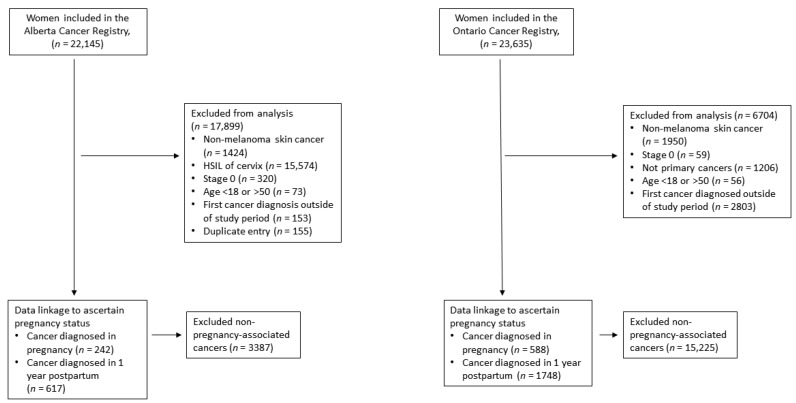
Study flow diagram.

Data on women aged 18–50 years, and with a primary cancer diagnosed between 2003 and 2015, were obtained from the Alberta Cancer Registry and the Ontario Cancer Registry. These registries systematically ascertain all cancer cases among children and adults in each province, where there is universal healthcare for all residents. Cases of nonmelanoma skin cancers, squamous intraepithelial neoplasia grade III (not invasive and not staged), high grade squamous intraepithelial lesion (*HSIL*) of the cervix or any other pre-invasive cases (Figure 1) were excluded. For women with multiple primary cancers, only the first cancer diagnosed in the study time period was considered.

Deterministic linkage, based on an individual’s universal health insurance number, sex and date of birth, was used to link data from each cancer registry to other health administrative databases in their respective province. Specifically, these other health administrative databases were used to identify livebirths, stillbirths and induced abortions (Appendix A). In Ontario, livebirths and stillbirths were ascertained from the ICES MOMBABY database, which includes data on all hospital-based deliveries in Ontario. Approximately 98% of births in Canada occur in hospital [7]. A validated algorithm, with >90% sensitivity, was used to ascertain induced abortions in the Discharge Abstract Database and the Ontario Health Insurance Plan (OHIP) claims database [8,9]. Ontario data were analyzed at ICES using a secure remote log-in using SAS version 9.4 (SAS Institute Inc., Cary, NC, USA). In Alberta, livebirths and stillbirths were jointly ascertained from the CIHI Discharge Abstract Database and the Alberta Perinatal Health Program. The Alberta Perinatal Health Program captures all livebirths and stillbirths at ≥20 weeks of gestation, either in hospital or at home if attended by a registered midwife. The same algorithm that was used in Ontario was used to ascertain induced abortions in the Discharge Abstract Database and Physician Claims Database in Alberta. Data analysis was conducted using SAS version 9.4 (SAS Institute Inc.) on the Alberta Health Services secure network. In both provinces, the date of a livebirth, stillbirth or induced abortion, and the gestational age at which this occurred, was used to estimate the date of conception. If gestational age was missing, it was imputed as nine weeks for an induced abortion, and 39 weeks for a livebirth or stillbirth. Gestational age was imputed for 98% of induced abortions and 3% of live/stillbirths. Women who had a cancer diagnosed during pregnancy or within 365 days following a birth or an induced abortion were considered to have a pregnancy-associated cancer. Cell sizes less than 6 were suppressed to prevent potential disclosure of subjects.

Descriptive statistics were used to characterize the population. The annual crude IR was calculated (with exact 95% confidence intervals (CI)) as the number of women with a pregnancy-associated cancer per 100,000 live or stillborn deliveries. As with estimates of maternal mortality, induced abortions were not included in the denominator. A sensitivity analysis was conducted to examine the crude IR excluding induced abortions from the numerator. Multifetal pregnancies were only counted once for both the numerator and denominator. In all cases, year was defined as the year that the birth or induced abortion occurred. For example, a woman diagnosed with a pregnancy-associated cancer in 2005, but who did not deliver until 2006, would be assigned to the year 2006. Women with a pregnancy-associated cancer were stratified into five-year age groups, and age standardized to the 2016 Canadian female population using direct standardization [10]. Women who were <20 or >49 years of age were excluded from this age-standardized approach, as the inclusion criteria for this study did not include all pregnancy-associated cancers diagnosed in women aged 15–19 and 50–54 years. The nonparametric test for trend, an extension of the Wilcoxon rank-sum test, was used to assess for significant temporal trends [11]. In Alberta, ethics approval for this study was obtained by the Health Research Ethics Board of Alberta–Cancer Committee at the University of Calgary; while in Ontario, the use of data for this study was permitted without review by a research ethics board under section 45 of the *Personal Health Information Protection Act*.

## 3. Results

Overall, 859 women in Alberta and 2336 women in Ontario were diagnosed with a pregnancy-associated cancer. The majority (71.8% in Alberta and 74.8% in Ontario) of pregnancy-associated cancers were diagnosed within one year postpartum. As seen in Table 1, while the demographic profile of women diagnosed with a pregnancy-associated cancer and survival following a pregnancy-associated cancer was comparable across both provinces, there was a statistically significantly higher proportion of women diagnosed with Stage 1 cancer in Alberta. Cancer stage was unknown for approximately half of all women with a pregnancy-associated cancer in Ontario. In both provinces, breast cancer was the most commonly diagnosed cancer during pregnancy, while thyroid cancer was the most commonly diagnosed cancer in the one-year postpartum period.

**Figure 2:**
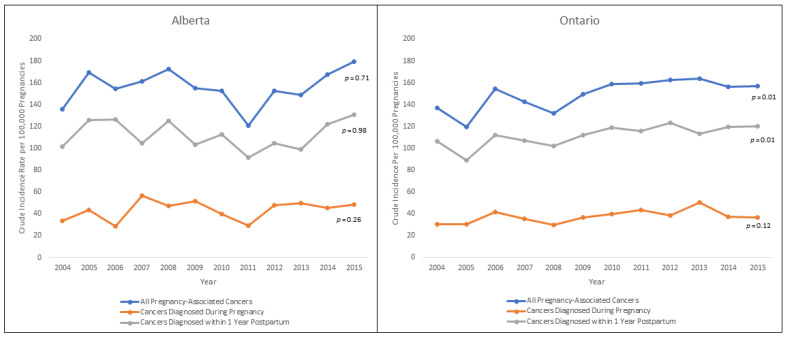
Temporal trends in the crude incidence rate (IR) of pregnancy-associated cancer in Alberta and Ontario, 2004–2015.

In Alberta, the crude IR of pregnancy-associated cancer was 156.2 per 100,000 deliveries (95% CI 145.8–166.7), and in Ontario, the IR was 149.4 per 100,000 deliveries (95% CI 143.3–155.4). When data on induced abortions were excluded, the crude IR of pregnancy-associated cancer resulting in a live or stillbirth was 153.1 per 100,000 deliveries (95% CI 142.8–163.5) in Alberta and 146.5 per 100,000 deliveries (95% CI 140.6–152.5) in Ontario. The age-standardized IRs among women aged 20–49 years were 189.7 (95% CI 178.0–201.3) and 223.3 (95% CI 215.8–230.8) per 100,000 deliveries in Alberta and Ontario, respectively. No temporal trend was seen for pregnancy-associated cancer in Alberta, but a statistically significant upward trend was seen in Ontario (Figure 2). The latter was largely driven by an increase in the IR of cancer diagnosed in the postpartum period.

## 4. Discussion

We observed crude IRs of pregnancy-associated cancer of 156.2 and 149.4 per 100,000 deliveries, respectively, in Alberta and Ontario. To our knowledge, these are the highest IRs of pregnancy-associated cancer reported in the literature [3,5,6,12,13]. We observed a temporal increase in the IR of pregnancy-associated cancer in Ontario, but not in Alberta. These results are similar to reports from two regions in Italy; while one region experienced a temporal increase in pregnancy-associated cancer between 2003 and 2015 [6], another region did not experience any change in a comparable time period (2001–2012) [13]. Studies from both Australia [3] and Denmark [5] have reported that the IR of pregnancy-associated cancer is increasing over time. Increases in pregnancy-associated cancer have been attributed to increasing maternal age [12,14]; however, Lee et al. observed that changes in maternal age only explained 14% of the increase in pregnancy-associated cancer in Australia [3]. Additionally, since studies of cancer incidence in the general population demonstrate increases in some types of cancer in young adults, there is a need to examine nonobstetrical-related population trends, such as obesity, smoking, and physical inactivity, as potential contributing factors [15,16].

The use of administrative data is both a strength and limitation of this study. All Canadian citizens have universal provincial health insurance coverage, meaning that the administrative and registry data used in this study cover the entire population and are not subject to selection bias. Additionally, cancer registries have legislated authority to collect data on cancer cases within their provincial jurisdictions. Moreover, administrative and registry data sources contain data that are consistently collected from a large number of subjects over a long time period, allowing researchers to explore temporal trends. A limitation of using administrative data herein relates to the lack of some clinical details which precludes a finer breakdown on cancer characteristics, treatments and long-term aspects of survivorship. We were unable to include spontaneous abortions due to poor ascertainment in administrative data; thus, our estimates of pregnancy-associated cancer are under-ascertained. Additionally, while data on women with cancer who had an abortion were available to the study team, we lacked data on abortions in women without cancer, and thus were not able to include this information in the denominator of our IR calculations. We attempted to overcome this limitation by including a sensitivity analysis that also excluded data on induced abortions in the numerator.

## 5. Conclusions

Women with pregnancy-associated cancer remain an under-studied group, yet they have unique needs and concerns during cancer treatment that persist into a likely long period of survivorship. The comprehensive and routine surveillance of temporal trends in the IR of pregnancy-associated cancer is an important first step in identifying the magnitude of the population impacted, while ongoing surveillance should be used to identify areas where additional resources are required to meet the health care needs of this population.

## Figures and Tables

**Table 1:** Characteristics of women with pregnancy-associated cancer in Alberta and Ontario, 2004–2015.

Characteristic	Alberta		Ontario	
	Cancer Diagnosed During Pregnancy (*n* = 242)	Cancer Diagnosed within 1 Year Postpartum (*n* = 617)	Cancer Diagnosed During Pregnancy (*n* = 588)	Cancer Diagnosed within 1 Year Postpartum (*n* = 1748)
Mean (SD) age at cancer diagnosis, years	31.8 (5.4)	31.8 (5.0)	31.8 (5.0)	32.7 (5.0)
Mean (SD) gestational age at birth, weeks	37.0 (3.4)	38.6 (2.2)	37.0 (3.4)	38.6 (2.2)
Preterm delivery (gestational age < 37 weeks)	28.9 (23.2–34.6)	8.9 (6.7–11.2)	29.6 (25.9–33.3)	8.8 (7.5–10.1)
Mean (SD) birthweight (grams)	3005.3 (731.8)	3305.0 (588.2)	2966.1 (761.1)	3356.5 (593.8)
Rural residence	9.5 (5.8–13.2)	13.0 (10.3–15.6)	8.0 (5.8–10.2)	8.1 (6.8–9.3)
Stage at cancer diagnosis				
1	31.4 (25.6–37.3)	41.2 (37.3–45.1)	20.4 (17.2–23.7)	24.9 (22.9–27.0)
2	16.1 (11.5–20.7)	12.5 (9.9–15.1)	15.3 (12.4–18.2)	10.8 (9.4–12.2)
3	11.6 (7.5–15.6)	9.4 (7.1–11.7)	11.1 (8.5–13.6)	7.9 (6.6–9.2)
4	7.0 (3.8–10.2)	7.1 (5.1–9.2)	2.6 (1.3–3.8)	4.9 (3.9–5.9)
Unknown or unstageable	33.9 (27.9–39.8)	30.3 (26.7–33.9)	50.7 (46.6–54.7)	51.4 (49.1–53.8)
Top-four cancer types				
Breast	24.0 (18.6–29.3)	17.8 (14.8–20.8)	27.7 (24.1–31.3)	20.5 (18.6–22.4)
Thyroid	18.2 (13.3–23.0)	23.3 (20.0–26.7)	20.2 (17.0–23.5)	34.2 (32.0–36.4)
Melanoma	11.2 (7.2–15.1)	8.3 (6.7–11.2)	13.6 (10.8–16.4)	10.5 (9.1–12.0)
Cervical	5.8 (2.8–8.7)	13.8 (11.1–16.5)	3.4 (1.9–4.9)	6.1 (4.9–7.2)
Mortality	12.3 (8.2–16.5)	11.7 (9.1–14.2)	14.8 (11.9–17.7)	13.6 (12.0–15.2)

All data are presented as %, 95% confidence interval (CI) unless otherwise indicated.

## Data Availability

Restrictions apply to the availability of these data. Data were obtained from the ICES Data Repository, Alberta Health, and Alberta Health Services. Individuals can make an independent request for data through these agencies.

## Appendix A

**Table A1:** Codes Used to Identify Study Population.

Condition	Code Used for Alberta Data	Code Used for Ontario Data
Nonmelanoma skin cancer	Cancer_site = ”Nonmelanoma Skin”	Curr_morph_cd = “80003” or “80103” or “80702” or “80703” or “80713” or “80812” or “80833” or “80903” or “80912” or “80913” or “80923” or “80933” or “80943” or “80973” or “80983” or “82473” or “84073” or “84103” or “88221” or “88323” or “91331” or “91403”
High grade squamous intraepithelial lesion (HSIL) of cervix	Cancer_site = “Cervix Uteri” and behaviour = 2; Morphology = “8077/2 Squamous intraepithelial neoplasia, grade III”	Curr_morph_cd = “80772”
Abortion	Discharge abstract database:Diagnosis code = International Classification of Disease (ICD) 10 code “O04” Physician claims: Diagnosis code = ICD 9 codes 635, 636, 637, 638	OHIP: feesuff= “A” and feecode = “S752” or “S785”
